# Correction: Does Human Migration Affect International Trade? A Complex-Network Perspective

**DOI:** 10.1371/journal.pone.0126879

**Published:** 2015-04-17

**Authors:** The PLOS ONE Staff

## Notice of Republication

This article was republished on April 6, 2015, due to the text of the original submission being published, rather than the text of the revised manuscript. The incorrect submission file was uploaded during the production process. Please download this article again to view the correct, revised version. The originally published, uncorrected article and the republished, corrected article are provided here for reference.

## Supporting Information

S1 Original Article Originally published, uncorrected article. (PDF)

S2 Revised Article Republished corrected article. (PDF)
