# Supplementary material for: Correction: Does Human Migration Affect International Trade? A Complex-Network Perspective
Source: PLoS One. 2015 Apr 17;10(4):e0126879. doi: 10.1371/journal.pone.0126879 (PMC4401537; doi:10.1371/journal.pone.0126879)
Supplement: S1 Original Article — (PDF) [file pone.0126879.s001.pdf]

# Does Human Migration Affect International Trade? A Complex-Network Perspective

Giorgio Fagiolo[1*], Marina Mastrorillo[2],

[1] Institute of Economics, Scuola Superiore Sant'Anna, Pisa, 56127, Italy, Email: g.fagiolo@sssup.it

[2] Princeton University, Princeton, New Jersey, 08540, USA, Email: mm28@princeton.edu

∗ Corresponding author

## Abstract

This paper explores the relationships between international human migration and merchandise trade using a complex-network approach. We firstly compare the topological structure of worldwide networks of human migration and bilateral trade over the period 1960-2000. Next, we ask whether the position of any pair of countries in the migration network affects their bilateral trade flows. We show that: (i) both weighted and binary versions of the networks of international migration and trade are strongly correlated; (ii) such correlations can be mostly explained by country economic/demographic size and geographical distance; (iii) pairs of countries that are more central in the international-migration network trade more. Our findings suggest that bilateral trade between any two countries is not only affected by the presence of migrants from either countries, but also by their relative embeddedness in the complex web of corridors making up the network of international human migration.

## Introduction

Cross-border human migration and international trade account for a large part of yearly mobility of people and goods across our planet, and their importance has been relentlessly growing during the last waves of globalization [1]. Over the period 1960-2010, for example, the share of total world exports over real-domestic product (GDP) has increased by 172%, whereas human migration, in terms of number of world immigrants, more than doubled, with an estimated migrant population of more than 200 million in 2010. Despite in the last decades governments have kept reducing barriers to trade without proportionally lowering those to immigration, also the share of world migrants to population has increased by almost 20%. The extraordinary growth in cross-country human migration and trade did not occur only intensively (i.e., increasing migration stocks over a fixed set of migration corridors), but also extensively (i.e., creation of new migration corridors). Indeed, over the period 1960-2000, the number of newly-created export channels between world countries exhibited a threefold increase. Similarly, simple back-of-the-envelope calculations on World Development Indicators (WDI) data [2], show that the number of new emigration corridors almost doubled.

Taken together, the intensive and extensive time evolution of international trade channels and migration corridors has led, over the years, to an intricate and complicated web of relationships between countries, whose properties have been recently investigated using a complex-network perspective [3]. The

topological properties of the International-Trade Network (ITN) [4–8] and the International-Migration Network (IMN) [9,10], and their evolution over time, have been explored with both a binary and weighted network approach [6,9,11]. Furthermore, their community structure [9,10,12,13] has been identified. Statistical null-network models [9, 14, 15] and economics-inspired gravity-like models have also been fitted to the data [16–18] in order to understand the determinants of the observed network regularities in each network.

A common feature of existing works on migration and trade networks is that they treat these two phenomena as completely independent. In other words, the topological properties of the ITN and IMN have been separately investigated as if migration and trade were two fully disconnected layers of the directed-weighted multi-graph where nodes are world countries and links represent their macroeconomic interaction channels (possibly including finance, investment, and other bilateral types of socio-economic macroeconomic relationships) [19]. It must be noted, however, that a large empirical literature in economics has deeply explored the causal connections between migration and trade. More specifically, several studies find quite a robust evidence suggesting that bilateral migration affects international-trade flows [20, 21]. As argued in Ref. [22], for example, trade between any two countries $(i, j)$ may be enhanced by the stock of immigrants present in either country and coming from the other one ($m_{ji}$ and $m_{ij}$). This is because migrants originating in $j$ and present in $i$ (and vice versa) may foster imports of goods produced in their mother country (bilateral consumption-preference effect) or reduce import transaction costs thanks to their better knowledge of both home- and host-country laws, habits, and regulations.

Note that such a *bilateral information effect* only takes into account the direct impact of migrants from either countries present in the other one to explain bilateral trade. However, one may posit that trade between any two countries can be fostered not only by bilateral-migration effects, but also thanks to migrants coming from other “third parties” and, more generally, by the overall connectivity and centrality of both countries in the IMN [23–25]. This is because the better a pair of countries is connected in the IMN, the larger the average number of third countries that they share as origin of immigration flows and the more likely the presence of strong third-party migrant communities in both countries. This may further enhance trade via both preference and information effects. Moreover, it may happen that two countries are relatively well connected in the IMN (in both binary and weighted terms) even if they share a very limited number of non-overlapping third parties. In such a case, one may ask whether a cosmopolitan environment engendered by the presence of many ethnic groups in both countries can be trade enhancing —and if so why.

Elaborating on these ideas, this paper expands upon existing literature in two respects. First, we compare the topological structure of the IMN and ITN and study their correlation patterns (see Ref. [26] for a complementary analysis that explores similar issues using a product-specific trade perspective). We also investigate the main determinants of these correlations and we find that economic and demographic country size, as well as geographical distance, play a key role in explaining differences and similarities between IMN-ITN topologies.

Second, we ask whether the position of countries in the IMN explains, in addition to bilateral-migration effects, their bilateral trade. We expand upon the existing literature by fitting gravity models of trade where country centrality is added as a further explanatory factor. We find that pairs of countries that

are more central in the IMN also trade more. Interestingly, we find that also inward third-party migrants coming from corridors that are not shared by the two countries can be trade enhancing, in addition to common inward ones. We suggest that this can be due to either learning processes of new consumption preferences by migrants whose origins are not shared by the two countries (e.g. facilitated by an open and cosmopolitan environment) or by the presence in both countries of second-generation migrants belonging to the same ethnic group.

# Materials and Methods

## Data and Definitions

Migration data employed in the paper come from the United Nations Global Migration Database [27], which comprises, for each year $y = \{1960, 1970, 1980, 1990, 2000\}$, an origin-destination square matrix recording bilateral migration between 226 countries. The generic element $(i, j)$ of each matrix is equal to the stock of migrants (corresponding to the last completed census round) originating in country $i$ and present in destination $j$, where migrant status is consistently defined in terms of country of birth.

As to merchandise trade, we employ the dataset provided by Kristian Gleditsch [28], which contains bilateral export-import yearly figures for the period 1950-2000. Trade matrices follow the flow of goods: rows represent exporting countries, whereas columns stand for importing countries. The generic bilateral element $(i, j)$ thus records exports from $i$ to $j$ in a given year. Trade figures, which are originally expressed in current US dollars, are then deflated to get real values.

We merged these two datasets by keeping, in each of the 5 years available in migration data, all countries that were present also in trade data with at least a positive import or export flow. This results in 5 origin-destination $N^y \times N^y$ matrices, where $N^y = \{109, 135, 158, 163, 183\}$. The sample of countries included explains more than 90% of total world trade flows and migration stocks in each year.

We employ additional country-specific data such as real gross domestic product (rGDP), population (POP) and per-capita real gross-domestic product (rGDPpc) from Penn World Tables version 6.3 (`https://pwt.sas.upenn.edu`). We also use bilateral country geographic, political and socio-economic data from the CEPII gravity dataset (see `http://www.cepii.fr`). The latter includes information about between-country geographical distance ($\delta$), contiguity (CONTIG, i.e. whether two countries share a border), existence of a preferential trade agreements (PTA), common language (COMLANG), etc. and will be mostly used to perform gravity-like exercises (more on this below).

We use trade and migration data to build a time sequence of 5 weighted-directed migration-trade (multi) graphs describing both bilateral-migration stocks and exports flows. More precisely, we define the international migration-trade network (IMTN) as a directed weighted multigraph wherein between any two nodes (countries) there can be at most four weighted-directed links, two of which describing bilateral export and the other two concerning bilateral migration. Alternatively, we can think to the IMTN as a time sequence of 2-layer weighted directed networks, the first layer representing the IMN and the second the ITN. In both cases, the IMTN at each time $y = 1960, \ldots, 2000$ is characterized by the pair of $N^y \times N^y$ weight matrices $(M^y, T^y)$, where $M^y$ and $T^y$ define respectively the weighted-directed

International Migration Network (IMN) and the weighted-directed International Trade Network (ITN). The generic element of $M^y$ represents the stock of migrants $m_{ij}^y$ originated in country $i$ and present at year $y$ in country $j$. Instead, the generic element of $T^y$ records the value of exports $t_{ij}^y$ from country $i$ to country $j$ in year $y$.

Accordingly, we define the binary projection of the IMTN through the pair of $N^y \times N^y$ adjacency matrices $(A_M^y, A_T^y)$, where the generic element of $A_X^y$, $X = \{M, T\}$, is equal to one if and only if the correspondent entry in $X^y$ is strictly positive (and zero otherwise).

Figure 1 plots the undirected weighted version of the IMN (a) and of the ITN (b) in year 2000. In the figures, link directions are suppressed to attain a better visualization of the graphs and only the top 5% of link weights are plotted. Link thickness is proportional to the logs of total bilateral migrants $(m_{ij}^y + m_{ji}^y)$ and the logs of total bilateral trade $(t_{ij}^y + t_{ji}^y)$, respectively. To get a feel of migration and trade determinants, node size is made proportional to the log of country population, while node color (from beige to red, i.e., from lighter to darker grey) represents logs of country rGDPpc (a measure of country income). The map allows one to appreciate some of the main general differences between IMN and ITN, e.g. the central role of Russia in the IMN (absent in the ITN) and the strong trade connections between the United States and South-Asian countries (absent in the IMN). Also, as expected, notice the widespread presence of low-income countries in the IMN (beige color), while the most relevant trade connections occur between countries with higher rGDPpc (red color).

## Comparative-Network Analysis

We begin with comparing the topological properties of the two layers of the IMTN. We compute basic statistics [29] to describe connectivity and asymmetry features of binary and weighted networks. More specifically, connectivity measures include: (i) network density; (ii) number of strongly and weakly connected components; (iii) undirected average path length. As far as binary-network asymmetry is concerned, we compute bilateral density, defined as the share of existing directed links that are reciprocated. Furthermore, we evaluate weighted asymmetry by computing the indicator studied in Ref. [30].

We also study the extent to which the two layers of the IMTN display any correlated behavior by exploring whether link weights $(m_{ij}^y, t_{ij}^y)$ are positively related, and why. A simple way to visualize any existing relation is to scatter plot link weights in the ITN vs IMN (on a log-log scale) in each year, where each dot represents, in the space $(m_{ij}^y, t_{ij}^y)$, an ordered pair of countries $(i, j)$ for which either $m_{ij}^y > 0$ or $t_{ij}^y > 0$. If a positive relation exists, natural candidates for explaining it are economic and demographic size (as proxied by rGDP and POP, respectively) and geographic distance $\delta_{ij}$ between the two countries. In particular, we rely on the the well-known empirical success of the gravity model for both migration and trade [31,32], which states that bilateral trade flows (respectively, migration stocks) are well explained by a gravity-like equation involving country sizes (rGDP and POP, respectively) and, inversely, geographical distance. If this is the case, one should expect that most of the variation in the cloud of points $(m_{ij}^y, t_{ij}^y)$ can be explained by larger products of country sizes and smaller distances. We check this hypothesis by giving to each dot in the scatter plots a size proportional to the product of country population divided by country distance ($\mathrm{POP}_i^y$*$\mathrm{POP}_j^y/\delta_{ij}$), and a color scale (from blue to red) depending on the product

of country rGDPs divided again by geographical distance (rGDP$_i$*rGDP$_j/\delta_{ij}$).

Next, we investigate matches and mismatches between ITN vs IMN binary structures. We want to assess, firstly, whether the presence/absence of directed migration corridors is correlated with the presence/absence of trade channels. We do so by comparing adjacency matrices $(A_M^y, A_T^y)$ and counting the percentage of total matches (either present or missing links), and the share of IMN links (respectively, ITN links) which are also present in the ITN (respectively, in the IMN). Secondly, we ask if rGDP, population and distances can explain matches and mismatches between binary structures. To answer this question, we assign in each year $y$ all possible $N^y(N^y-1)$ pair of countries to one of the four possible cases as far as presence/absence of a link in the two layers of the IMTN is concerned, namely: $(C_1)$ no link in both IMN and ITN; $(C_2)$ link in ITN and no link in the IMN; $(C_3)$ link in IMN and no link in the ITN; $(C_4)$ link in both ITN and IMN. Therefore, in each year $y$ we set up a partition of all possible $N^y(N^y-1)$ directed edges in four subsets $(s_1^y, s_2^y, s_3^y, s_4^y)$, where subset $s_h^y$ contains all directed edges that satisfy case $C_h$. We then compute, for each year separately, average and standard deviation of the quantities $q_{ij}^y = \log(\text{rGDP}_i^y) \cdot \log(\text{rGDP}_j^y)$ and $\log(\delta_{ij})$ over each separate subset $s_h^y$. As a result, the time-sequence of subsets $\{s_h^y\}$, where $h = 1, \ldots, 4$ and $y = 1960, \ldots, 2000$, can be characterized by four coordinates, i.e. conditional average and standard deviation of $q_{ij}^y$ and $\log(\text{rGDP}_j^y)$. To simplify things, we collapse standard deviations into one variable, defined as the product of standard deviations of $q_{ij}^y$ and $\log(\text{rGDP}_j^y)$. Each of all possible 20 subsets (number of classes times number of years) can then be visualized in a scatter plot whose x- and y-axis feature the mean of $q_{ij}^y$ and $\log(\text{rGDP}_j^y)$, respectively. Each dot can then be characterized by a color representing its class, a size proportional to the product of standard deviations, and a label identifying the year. This would eventually allow one to investigate if dots of different classes exhibit different patterns as far as size and distance is concerned, and if dots of consecutive years are sufficiently close to each other once within-class conditional standard deviation is properly taken into account.

Finally, we study correlation patterns of node-network statistics between the two layers of the IMTN in both their binary and weighted representations. For each layer separately, we compute node in- and out-degrees and strengths [33], average nearest-neighbor degrees and strengths [34], binary and weighted clustering coefficients [35, 36] and a number of binary and weighted node-centrality indicators, ranging from Bonacich [37,38] and Page-Rank [39] centrality, to hubs and authority scores [40]. As it is customary in the literature [7, 9], we compute weighted statistics using the logs of IMN and ITN link weights. We then ask whether countries that are more connected, clustered or central in the IMN layer are also more connected, clustered or central in the ITN. We also explore whether country-size may drive any emerging correlation (e.g. countries may be more connected, clustered or central in both layers just because their are larger) by adding information on country rGDP and POP to country-specific network indicators in ITN-IMN scatter plots.

## Panel Regressions

In addition to correlation patterns, we study whether network effects are present in the causal link going from migration to trade. We want to test if bilateral trade between any two countries is enhanced the

more: (i) these two countries share migrants between themselves (direct bilateral effect); (ii) they are jointly more central in the IMN. In particular, we aim to check if having more inward connections or receiving more immigrants boots bilateral trade. This can happen either from inward channels shared with the other country (common inward effect) or through non-overlapping ones (non-overlapping inward effect).

We explore these issues by performing a set of econometric exercises using a standard gravity-model of trade [31], expanded to take into account migration network effects. Building on Ref. [41], we fit to our data a gravity model whose general specification reads:

$$\log \tau_{ij}^y = \kappa + \phi_i^y + \gamma_j^y + \alpha \log(\delta_{ij}) + \boldsymbol{\beta} \boldsymbol{Z}_{ij}^y + \boldsymbol{\mu} \boldsymbol{W}_{ij}^y + \varepsilon_{ij}^y \tag{1}$$

where $\varepsilon_{ij}^y$ is the error term; $\kappa$ is a constant; $\tau_{ij}^y = t_{ij}^y + t_{ji}^y$ is total bilateral trade; $(\phi_i^y, \gamma_j^y)$ are country-time importer-exporter dummies controlling for all country-specific variables such as rGDP and POP (defined as $\phi_i^y = 1$, resp. $\gamma_j^y = 1$, if country $i$ (resp. $j$) is the importer, resp. the exporter, and zero otherwise.); $\delta_{ij}$ is geographical distance; $\boldsymbol{Z}_{ij}^y$ features bilateral country dummies (CONTIG, COMLANG, PTA$^y$); and $\boldsymbol{W}_{ij}^y$ is a vector of migration-related network variables accounting for bilateral and common vs. non-overlapping indirect effects. Note that all our results are robust to additional controls such as common religion, common colonial ties, and landlocking effects.

We separately test five different econometric specifications to check for alternative hypotheses about how network variables affect bilateral trade. In the first one, we only control for baseline gravity-related variables ($\log(\delta_{ij})$, CONTIG, COMLANG, PTA$^y$), i.e. $\boldsymbol{W}_{ij}^y$ does not appear. The second specification augments the first one by including in $\boldsymbol{W}_{ij}^y$ only total bilateral migration stock, defined as $\text{BIL\_MIG}_{ij}^y = \log(m_{ij}^y) + \log(m_{ji}^y)$.

In the remaining three specifications, we add also network, commong and non-overlapping, effects related to country inward centrality in the IMN. We distinguish between binary and weighted centrality indicators, to understand the role played by extensive migration margins (i.e. the number of inward corridors) and intensive migration margins (i.e. the stock of immigrants). For the binary case, we employ as a measure of country centrality *in-degree country centralization*, defined as:

$$\text{IN\_CENTR}_i^y = \frac{ind_i^y}{N^y} \tag{2}$$

where $ind_i^y$ is country in-degree (i.e. the number of inward links of country $i$). We use inward corridors only because we expect inward migration to be relevant in explaining bilateral trade rather than outward channels. Notice that in-degree centralization is highly and positively correlated with all other (binary and weighted) centrality indicators in the IMN (i.e. eigenvector-based indicators, betweenness centrality, etc.). For this reason, our results are quite robust to alternative centrality measures.

Since we employ importer-exporter time dummies, in the third specification, we add the log of the sum of country $i$ and $j$ in-degree centralization:

$$\text{IN\_CENTR}_{ij}^y = \text{IN\_CENTR}_i^y + \text{IN\_CENTR}_j^y, \tag{3}$$

instead of the two separately.

Furthermore, we study the role of third-party (indirect) common and non-overlapping inward migration channels. To do so, the fourth specification features only the log of the share of common in-neighbors of any given pairs of countries ($\text{COMM\_IN}_{ij}^{y}$), whereas, in the fifth and final specification, we control for both $\text{COMM\_IN}_{ij}^{y}$ and the log of the share of inward channels that the two countries do not share ($\text{NOTCOMM\_IN}_{ij}^{y}$), where shares are computed dividing by $N^y$. In other words, given any two countries $i$ and $j$, we count directed links pointing to $i$ and $j$ originating from third countries $h$ that send migrants to both $i$ and $j$. The residual contribution accounts for the number of inward corridors originated from third countries $k$ that only send migrants to either $i$ or $j$.

In the weighted case, we explicitly consider link weights in the IMN (i.e. logs of migrant stocks). We then replace in Eq. (3) country in-degrees ($ind_i^y/N^y$) with country in-strength ($ins_i^y/V^y$), where now we re-scale in-strength by the volume of the network in year $y$ (i.e., total sum of logged migrant stocks). We compute $\text{COMM\_IN}_{ij}^{y}$ by summing up the weights of commonly-shared inward channels. Similarly, $\text{NOTCOMM\_IN}_{ij}^{y}$ is obtained by summing up link weights over all inward links originated from third countries $k$ that only send migrants to either $i$ or $j$.

Estimation of Eq. (1) can be plagued by endogeneity issues. Indeed the error term may be correlated with the explanatory variables due to a reverse-causation link going from trade to migration. This is true whenever we use $\text{BIL\_MIG}_{ij}^{y}$ as a regressor, but also when we augment the equation with *weighted* network-related variables. We argue that this problem may be almost irrelevant in terms of binary network variables that count migration corridors only, as it is very unlikely that changes in bilateral-trade levels may destroy or form new links in the IMN. When link weights in the IMN are used to compute country in-centrality, however, it may well be the case that changes in bilateral trade may also impact on migration stocks and in turn on country centrality. To solve this problem we employ a standard instrumental-variable (IV) approach. Borrowing from Refs. [42, 43], we set up an auxiliary streamlined gravity regression to instrument bilateral migration stocks $m_{ij}^y$. More formally, we use ordinary least-squares (OLS) to fit to the data the following specification:

$$\mu_{ij}^y = \log(m_{ij}^y) = \varsigma_i^y + \zeta_j^y + \alpha \log(\delta_{ij}) + \nu_{ij}^y, \tag{4}$$

where ($\varsigma_i^y, \zeta_j^y$) are origin and destination country-time dummies and $\nu_{ij}^y$ is a white-noise error. The specification in Eq. (4), as expected, is able to explain almost 75% of variation in bilateral stocks. Furthermore, according to the F test (test statistic = 12.685), the employed instruments are valid.

Next, we use the predictions $\hat{\mu}_{ij}^y$ of the model in Eq. (4) to replace $\log(m_{ij}^y)$ and $\log(m_{ji}^y)$ in the definition of $\text{BIL\_MIG}_{ij}^{y}$. Furthermore, in order to instrument weighted centralization indicators, we employ $\hat{\mu}_{ij}^y$ to build, in each year $y$, a predicted logged IMN weighted matrix $\log \hat{M}^y = \{\hat{\mu}_{ij}^y\}$. Notice that since we use logs of $m_{ij}^y$ in Eq. (4), we automatically fit only strictly-positive migration stocks, i.e. we only consider non-zero weights. Therefore, predicted IMN weighted matrices are characterized by a binary projection $\hat{A}^y$ that is exactly equal to the observed one in each year, i.e. $\hat{A}_M^y = A_M^y$. We then employ predicted weighted IMN matrices to re-compute $\text{IN\_CENTR}_{ij}^{y}$, $\text{COMM\_IN}_{ij}^{y}$ and $\text{NOTCOMM\_IN}_{ij}^{y}$ and use them as instrumented regressors in Eq. (1), which we estimate using OLS. Similar results are obtained

with Poisson pseudo maximum-likelihood estimation [44].

# Results and Discussion

## ITN vs. IMN: Descriptive Statistics

We begin with a comparison between the topological properties of the two IMTN layers across time. Table 1 reports for the years 1960, 1980 and 2000 the main features of the two networks. Note that both networks are extremely dense. The ITN increased its density by 50% during the period covered by our data, and became more dense than the IMN in 2000. As expected, the ITN is also more symmetric than the IMN, as testified by a larger bilateral density (i.e. the percentage of reciprocated directed links). This is because a trade channel is easier to reciprocate than a migration corridor. This is true also when one takes into account the weights of the links: weighted asymmetry [30] is indeed larger in the IMN, capturing the fact that countries tend to be more bilaterally balanced in trade than in migration. Note also that both networks are always weakly and (almost) strongly-connected. Indeed, the number of weakly connected components is always one and strong connectivity is not achieved before year 2000 only because of the presence of one or two (strongly) not-connected countries, typically small and peripheral nations. Finally, as already noticed in Refs. [7,9], the IMN features a more marked small-world property, with average-path lengths smaller than in the ITN.

## ITN vs. IMN: Correlation patterns

We now study the extent to which the two layers of the IMTN display any correlated behavior. In what follows, we illustrate our findings for year 2000 only. However, similar results consistently hold also for the other years in the sample.

We start exploring whether link weights $(m_{ij}^y, t_{ij}^y)$ are positively related, and why. Figure 2 shows a scatter of link weights in the ITN vs IMN (log scale) in year 2000. Note first how a stronger link-weight in the ITN is typically associated to a stronger migration link-weight: if $i$ exports a higher trade value to $j$, in $j$ there is also a larger stock of migrants originated in $i$.

Figure 2 also suggests that most of the variation in the cloud of points $(m_{ij}^y, t_{ij}^y)$ is indeed explained by larger country sizes and smaller distances in a gravity-like fashion: red and large dots (higher values for $\text{POP}_i^y{*}\text{POP}_j^y/\delta_{ij}$ and $\text{rGDP}_i{*}\text{rGDP}_j/\delta_{ij}$) are located in the north-east part of the plot. This is more evident for the relation between trade, rGDP and $\delta$, than in the case of migration. Indeed, there exist large (and red) dots characterized by high trade values but relatively low migration stocks. This is the case of migration of Chinese people to India, which is historically feeble, unlike correspondent exports flows. Similarly, there are large dots associated with intermediate trade levels and very high migration stocks. These refer to the triangle Bangladesh, India and Pakistan, which experienced huge migration flows at the time of partitioning of India.

We move now to a comparison of ITN and IMN binary topologies. Results are presented in Figure 3. Two main findings stand out. First, the two networks have become more and more similar in terms

of presence/absence of links. Second, this has happened thanks to an increasing number of migration corridors that became also trade channels. On the contrary, the share of trade channels that are also migration corridors remained constant and even declined. Notice that all these shares are statistically larger than the expected ones obtained under null models in which the in- and out-degree sequence is kept fixed and links are accordingly reshuffled [45]. A possible explanation for the patterns in Figure 3 lies in the joint effect on the ITN of a relatively small initial density (as compared to the IMN) and the massive lowering of barriers occurring in world trade in the second half of the last century. Another possibility is the existence of a causal link from migration to trade, which we shall explore in more details below in our regression exercises.

To see if real GDP and distances can also explain matches and mismatches between binary structures, we plot for each year the averages of the quantities $q^y_{ij} = \log(\mathrm{rGDP}^y_i)\cdot\log(\mathrm{rGDP}^y_j)$ and $\log(\delta_{ij})$, conditional to the four possible cases (depicted with different colors), namely: (i) no link in both IMN and ITN (red); (ii) link in ITN and no link in the IMN (green); (iii) link in IMN and no link in the ITN (blue); (iv) link in both ITN and IMN (magenta), see Figure 4. It is easy to see that a simultaneous absence vs presence of a link is due to the combination of, respectively, low rGDPs and high distances vs high rGDPs and short distances. Furthermore, as expected, the IMN is more sensible to distance than the ITN: a link in the ITN that is not present in the IMN is typically associated to large distances. On the contrary, the ITN is more sensible to rGDP. Even at smaller distances, country size plays a difference: when the latter is small enough, links in the IMN tend not to appear in the ITN. Note also that these results are very robust across time (all same-color dots are very close to each other) and display quite a good precision (cf. the relatively small conditional dispersion, i.e. colored balls do not overlap). Similar findings are obtained when rGDP is replaced by country population.

We now contrast ITN and IMN layers in terms of node network statistics. For the sake of brevity, we only show results related to: (i) total degree: the sum of inward and outward links of a node; (ii) total strength: sum of inward and outward link weights of a node; (iii)-(iv) total average nearest-neighbor degree (ANND) and strength (ANNS): average of node degree (respectively, strength) of the neighbors of a node, no matter the directionality of the links held by the node. Whereas total degree and ANND are computed on the binary IMTN, node strength and ANNS employ its weighted representation. Similar findings hold for the whole range of network statistics that we have computed, including binary/weighted clustering and centrality indicators.

Figure 5 shows that both node degrees and strengths are positively and linearly related in the two layers, see panels (a) and (c). This means that if a country has more trade channels (respectively, trades more), it also carries more migration channels (respectively, holds larger immigrant/emigrant stocks). Again, it is easy to see that this positive relation is mostly explained by country demographic and economic size. We also find that if a country trades with countries that either trade with many other partners or trade a lot, is also connected to countries that hold a lot of migration channels or stocks, i.e. both ANND and ANNS are positively correlated in the two layers, cf. panels (b) and (d).

However, unlike what happens for degrees and strength, smaller levels of ANND and ANNS in the IMTN are associated to larger demographic and economic country sizes. To see why this is the case, we study binary and weighted disassortativity patterns *within* the two IMTN layers. Figure 6 scatter-

plots node total degree (respectively, strength) vs ANND (respectively, ANNS), separately for ITN and IMN, and correlates this information with country population and rGDP as in Figure 5. As already known [7, 9, 46], both networks display a marked (binary and weighted) disassortative behavior: the partners of more strongly connected nodes are weakly connected. However, larger countries (i.e. with higher levels of rGDP and POP) also hold larger degrees and strengths. Therefore, countries with larger levels of ANND and ANNS are smaller, in both economic and demographic terms.

## Does Migration Affect Trade?

In the preceding sections, we have explored the patterns of correlation between the two layers of the ITMN and their determinants. We move now to assessing whether there exists any causal relationship between the IMN and the ITN. As we have already noted when discussing the evidence on binary structures (see Figure 3), the emergence of links in the ITN seems to be driven by existing migration corridors. More generally, we want to test if (as already found in several papers, see e.g. Ref. [22]) bilateral trade between country $i$ and $j$ is boosted by the presence of migrants in $i$ coming from $j$ and vice versa (direct bilateral effect). Furthermore, we want to understand if indirect network effects may play a role in enhancing bilateral trade. Our hypothesis is that bilateral trade may increase the more the two countries under consideration are inward central in the IMN. This may happen either because they share common immigration corridors or attract more immigrants from common origins or because they are more inward connected (in both intensive and extensive terms) with non-overlapping origins.

To test these hypotheses, we estimate Eq. (1) using our migration and trade data. We separately perform two sets of exercises, one when binary network indicators are considered and one when country centrality is measured using weighted statistics. In each exercise, we estimate the five specifications discussed above. Whenever on the right-hand side of the regression either $\mathrm{BIL\_MIG}_{ij}^{y}$ or weighted centrality indicators do appear, we instrument them using Eq. (4) and the procedure explained in the Materials and Methods section.

Regression results are reported in Tables 2 (binary centrality indicators) and 3 (weighted centrality indicators). Note first that all specifications attain a very high goodness of fit, as it always happens in empirical gravity estimation. Notice that the residuals of the regression specifications where we instrumented migration stocks are not correlated with the instruments, indicating that the latter are actually exogenous. The addition of network statistics induces an increase in adjusted $R^2$, albeit limited. The impact of distance, contiguity, common language and participation to a trade agreement are strong, significant, and signed in line with existing studies. Total bilateral migration positively affects bilateral trade as expected, and its impact is almost constant no matter the chosen specification [41].

In both tables, columns (3)-(5) report regressions where country-network centrality indicators are accounted for. We find that the more total inward-migration corridors and immigrants a pair of country holds, the larger their bilateral trade, i.e. $\mathrm{IN\_CENTR}_{ij}^{y}$ has a positive and significant effect on trade in both extensive and intensive terms.

To check whether this is due to common vs non-overlapping in-neighboring channels, columns (4) and (5) report specifications where only $\mathrm{COMM\_IN}_{ij}^{y}$ or both $\mathrm{COMM\_IN}_{ij}^{y}$ and $\mathrm{NOTCOMM\_IN}_{ij}^{y}$ enter

the model. Estimates suggest that: (i) common third parties and stocks of immigrants coming from common origins have a positive effect on bilateral trade; (ii) once one controls for common third-party effects (either binary or weighted), the number of non-overlapping channels or the stock of immigrants originating from non-common third parties are also trade enhancing, even if with a smaller impact.

As a robustness check, notice that all the results hold true also if country-time importer-exporter dummies are removed and replaced with country rGDPs. Furthermore, similar results are obtained if we employ $t_{ij}^{y}$ as dependent variable and we separately add as regressors country-centrality indicators (IN_CENTR$_{i}^{y}$ and IN_CENTR$_{j}^{y}$). Note that the positive effect on trade of NOTCOMM_IN$_{ij}^{y}$ is preserved when one enters this variable in the regressions without COMM_IN$_{ij}^{y}$.

The foregoing evidence suggests that in addition to bilateral-migration effects, trade between any two countries $(i, j)$ may increase due to their binary and weighted connectivity in the IMN. This might happen via two related mechanisms. First, pairs of countries holding more inward links or more immigrants are more likely to share an increasing number of inward corridors and/or immigrants coming from common third-party migration origins $k \neq (i, j)$ and therefore, thanks to consumption-preference and information effects, more bilateral trade [23–25]. Second, a smaller but still significant trade-enhancing effect can come from the presence in both countries of a higher number of inward migration corridors that are however not shared by $i$ and $j$ and larger stocks of immigrants coming from such corridors. In other words, if countries $i$ and $j$ host migrants originated respectively from countries $I = \{i_1, \ldots, i_m\}$ and $J = \{j_1, \ldots, j_n\}$, with $I \bigcap J = \emptyset$, the larger $m$ and/or $n$, and the higher the stock of migrants originating in such countries, the higher bilateral trade between the two countries. This second trade-enhancing effect can have a twofold explanation. On the one hand, more immigrants coming from non-overlapping migration channels, coupled with commonly-shared origins, may imply more cosmopolitan and inclusive environments in both countries, which may in turn foster, in all ethnic groups, learning processes about consumption patterns of ethnic groups commonly shared by the two countries, and therefore more bilateral trade. On the other hand, more immigrants arrived through non-overlapping inward migration channels imply a higher probability to find in both countries more second-generation migrants belonging to the same ethnic group. Indeed, our data record migrants according their birth-place and not necessarily their ethnic origin. Therefore, it may be the case that, even if countries $h_i$ and $h_j$ are not shared as inward channels by $i$ and $j$ respectively, they can send second-generation migrants belonging to the same ethnic group to $i$ and $j$, thus enhancing their bilateral trade. This effect cannot be entirely picked up by COMM_IN$_{ij}^{y}$ and it can thus show up, as Tables 2 and 3 suggest, in the binary and weighted coefficient of NOTCOMM_IN$_{ij}^{y}$.

# Conclusions

This paper has explored the relationships between international migration and trade using a complex-network approach. More specifically, we have performed two related exercises. First, we have investigated the patterns of correlation between the ITN and the IMN, comparing link weights, topological structures and node network statistics. We have found that trade and migration networks are strongly correlated and such relation can be mostly explained by country economic and demographic size and geographical

distance. Second, we have asked whether country centrality in the IMN can explain bilateral trade. Expanding upon the existing economic literature, we have fit to the data gravity models of bilateral trade adding migration-network variables among the regressors. These control for country inward centralization, and the number and intensity of common vs. non-overlapping inward migration channels. Our results indicate that the larger the number and the intensity of inward —both common and non-overlapping— migration corridors held by any two countries, the higher bilateral trade.

More generally, our work suggests two main avenues along which the study of macro-economic networks, where nodes are world countries and links represent their possible interaction channels along many economic and social dimensions, can be fruitfully pursued. First, one might build a multi-graph representation of the macroeconomic network, where between any two countries there may exist many links, each representing a different type of between-country interaction (trade, mobility, finance, foreign investment, etc.). This may allow one to explore whether different layers display similar topological properties, and whether such properties are correlated, or causally linked, between layers. Second, our work is a modest step towards a better understanding of how the properties of a network can influence how its nodes behave and perform over time [47,48]. Once possible endogeneity issues are properly taken into account, this empirical research program may convey new and interesting insights on the importance of network structure in shaping aggregate dynamics of the societies and economies where we live.

## Acknowledgments

Thanks to Massimo Riccaboni, Rodolfo Metulini and all the people at IMT (Lucca, Italy) for their helpful comments.

## References

1. National Research Council (2010). Understanding the changing planet: Strategic directions for the geographical sciences. URL `http://www.nap.edu`.
2. The World Bank Group (2013). World development indicators (WDI) online. URL `http://publications.worldbank.org/WDI/`.
3. Schweitzer F, Fagiolo G, Sornette D, Vega-Redondo F, Vespignani A, et al. (2009) Economic networks: The new challenges. Science 325: 422–425.
4. Serrano A, Boguñá M (2003) Topology of the World Trade Web. Physical Review E 68.
5. Garlaschelli D, Loffredo M (2004) Fitness-dependent topological properties of the World Trade Web. Physical Review Letters 93.
6. Fagiolo G, Schiavo S, Reyes J (2008) On the topological properties of the World Trade Web: A weighted network analysis. Physica A 387: 386873.
7. Fagiolo G, Schiavo S, Reyes J (2009) World-Trade Web: Topological properties, dynamics, and evolution. Physical Review E 79.
8. Fagiolo G, Schiavo S, Reyes J (2010) The evolution of the World Trade Web: A weighted- network approach. Journal of Evolutionary Economics 20: 479-514.

9. Fagiolo G, Mastrorillo M (2013) International Migration Network: Topology and modeling. Physical Review E 88: 012812.

10. Davis KF, D'Odorico P, Laio F, Ridolfi L (2013) Global Spatio-Temporal Patterns in Human Migration: A Complex Network Perspective. PLoS ONE 8: e53723.

11. Garlaschelli D, Loffredo M (2005) Structure and evolution of the World Trade Network. Physica A 355: 138-44.

12. Barigozzi M, Fagiolo G, Mangioni G (2011) Identifying the community structure of the international-trade multi-network. Physica A: Statistical Mechanics and its Applications 390: 2051 - 2066.

13. Piccardi C, Tajoli L (2012) Existence and significance of communities in the World Trade Web. Physical Review E 85: 066119.

14. Squartini T, Fagiolo G, Garlaschelli D (2011) Randomizing World trade. i. a binary network analysis. Physical Review E 84: 046117.

15. Squartini T, Fagiolo G, Garlaschelli D (2011) Randomizing World trade. ii. a weighted network analysis. Physical Review E 84: 046118.

16. Fagiolo G (2010) The international-Trade Network: gravity equations and topological properties. Journal of Economic Interaction and Coordination 5: 1-25.

17. Duenas M, Fagiolo G (2013) Modeling the international-Trade Network: a gravity approach. Journal of Economic Interaction and Coordination 8: 155-178.

18. Ward MD, Ahlquist JS, Rozenas A (2013) Gravity's rainbow: A dynamic latent space model for the World Trade Network. Network Science 1: 95–118.

19. Schiavo S, Reyes J, Fagiolo G (2010) International trade and financial integration: a weighted network analysis. Quantitative Finance 10: 389-399.

20. Gaston N, Nelson D (2011) International migration. In: Bernhofen D, Falvey R, Greenaway D, Kreickemeier U, editors, Handbook of International Trade, London: Palgrave.

21. Egger PH, von Ehrlich M, Nelson DR (2012) Migration and trade. The World Economy 35: 216-241.

22. Gould DM (1994) Immigrant links to the home country: Empirical implications for u.s. bilateral trade flows. The Review of Economics and Statistics 76: 302-16.

23. Rauch JE, Trindade V (1999) Ethnic chinese networks in international trade. NBER Working Papers 7189, National Bureau of Economic Research, Inc. URL `http://ideas.repec.org/p/nbr/nberwo/7189.html`.

24. Felbermayr GJ, Jung B, Toubal F (2010) Ethnic networks, information, and international trade: Revisiting the evidence. Annals of Economics and Statistics 10: 41-70.

25. Felbermayr GJ, Toubal F (2012) Revisiting the trade-migration nexus: Evidence from new OECD data. World Development 40: 928 - 937.

26. Sgrignoli P, Metulini R, Schiavo S, Riccaboni M (2013) The relation between global migration and Trade Networks. physics.soc-ph 1310.3716v2, arXiv.org.

27. Ozden C, Parsons CR, Schiff M, Walmsley TL (2011) Where on earth is everybody? the evolution of global bilateral migration 1960-2000. World Bank Economic Review 25: 12-56.

28. Gleditsch K (2002) Expanded trade and GDP data. Journal of Conflict Resolution 46: 712–24.

29. Newman M (2010) Networks: An Introduction. Oxford University Press, USA.

30. Fagiolo G (2006) Directed or undirected? a new index to check for directionality of relations in socio-economic networks. Economics Bulletin 3: 1–12.

31. van Bergeijk P, Brakman S, editors (2010) The Gravity Model in International Trade. Cambridge University Press, Cambridge.

32. Lewer JJ, Van den Berg H (2008) A gravity model of immigration. Economics Letters 99: 164-167.

33. Barthélemy M, Barrat A, Pastor-Satorras R, Vespignani A (2005) Characterization and modeling of complex weighted networks. Physica A 346: 34–43.

34. Barrat A, Barthélemy M, Pastor-Satorras R, Vespignani A (2004) The architecture of complex weighted networks. Proceedings of the National Academy of Sciences 101: 3747-52.

35. Saramaki J, Kivelä M, Onnela J, Kaski K, Kertész J (2007) Generalizations of the clustering coefficient to weighted complex networks. Physical Review E 75: 027105.

36. Fagiolo G (2007) Clustering in complex directed networks. Physical Review E 76: 026107.

37. Bonacich P (1987) Power and Centrality: A Family of Measures. American Journal of Sociology 92: 1170–1182.

38. Bonacich P, Lloyd P (2001) Eigenvector-like measures of centrality for asymmetric relations. Social Networks 23: 191–201.

39. Brin S, Page L (1998) The anatomy of a large-scale hypertextual web search engine. In: Proceedings of the seventh international conference on World Wide Web. Amsterdam, The Netherlands: Elsevier Science Publishers, WWW7, pp. 107–117.

40. Kleinberg J (1999) Authoritative sources in a hyperlinked environment. Journal of the ACM 465: 604–632.

41. Parsons CR (2012) Do migrants really foster trade ? the trade-migration nexus, a panel approach 1960-2000. Policy Research Working Paper Series 6034, The World Bank.

42. Romer DH, Frankel JA (1999) Does trade cause growth? American Economic Review 89: 379-399.

43. Ortega F, Peri G (2011) The aggregate effects of trade and migration: Evidence from OECD countries. IZA Discussion Papers 5604, Institute for the Study of Labor (IZA). URL `http://ideas.repec.org/p/iza/izadps/dp5604.html`.

44. Silva JMCS, Tenreyro S (2006) The log of gravity. The Review of Economics and Statistics 88: 641-658.

45. Maslov S, Sneppen K (2002) Specificity and Stability in Topology of Protein Networks. Science 296: 910-913.

46. Garlaschelli D, Loffredo M (2005) Structure and evolution of the World Trade Network. Physica A 355: 138–44.

47. Chinazzi M, Fagiolo G, Reyes JA, Schiavo S (2013) Post-mortem examination of the International Financial Network. Journal of Economic Dynamics and Control 37: 1692-1713.

48. Ductor L, Fafchamps M, Goyal S, van der Leij M (2013). Social Networks and Research Output. Review of Economics and Statistics, forthcoming.

# Figure Legends

**Figure 1.** The International-Migration Network (a) and the International-Trade Network (b) in year 2000. The figure plots the undirected weighted version of the ITN and IMN where only top 5% of bilateral link weights (total number of bilateral trade and total number of bilateral migrants) are drawn. Tickness of links in the plot is proportional to the logs of link weights. Node size is proportional to the log of country population. Node color represents country income (rGDPpc), from beige (low-income countries) to red (high-income countries).

**Figure 2.** IMN vs ITN link weights. Logarithmic scale. Markers size is proportional to the log of $\text{POP}_i^y$*$\text{POP}_j^y/\delta_{ij}$. Colors scale (blue to red) is from lower to higher values of logs of $\text{rGDP}_i^y$*$\text{rGDP}_j^y/\delta_{ij}$. Year=2000.

**Figure 3.** IMN vs ITN: Comparison of binary structure. Tot Matches: % of total matches (either missing or present links). IMN Links in ITN: % of IMN links which are also present in the ITN. ITN Links in IMN: % of ITN links which are also present in the IMN.

**Figure 4.** Scatter plot of average $\log(\text{rGDP}_i^y) \cdot \log(\text{rGDP}_j^y)$ versus average $\log(\delta_{ij})$ conditional on matches/mismatches between IMN vs ITN binary structures. Colors: Red=Absence of link in both ITN and IMN. Green=No link in IMN, link in ITN. Blue=No link in ITN, link in IMN. Magenta=Link in both ITN and IMN. Marker size is proportional to the product of standard deviations of $\log(\text{rGDP}_i^y) * \log(\text{rGDP}_j^y)$ and $\log(\delta_{ij})$, conditional to matches/mismatches between IMN vs ITN binary structures.

**Figure 5.** Correlation of node network statistics between IMN and ITN in year 2000. (a) Total degree; (b) Average nearest-neighbor degree (ANND); (c) Total strength; (d) Average nearest-neighbor strength (ANNS). Marker size is proportional to logs of $\text{POP}_i^y$. Colors scale (blue to red) is from lower to higher values logged $\text{rGDP}_i^y$.

**Figure 6.** Disassortativity patterns within IMN and ITN in year 2000. Marker size is proportional to logs of $\text{POP}_i^y$. Colors scale (blue to red) is from lower to higher values logged $\text{rGDP}_i^y$.

# Tables

| | 1960 | | 1980 | | 2000 | |
|---|---|---|---|---|---|---|
| | ITN | IMN | ITN | IMN | ITN | IMN |
| No. Nodes | 109 | 109 | 158 | 158 | 183 | 183 |
| Density | 0.3843 | 0.5808 | 0.4628 | 0.5080 | 0.5687 | 0.5503 |
| Bilateral Density | 0.8439 | 0.7234 | 0.8697 | 0.6975 | 0.9802 | 0.7097 |
| Weighted Asymmetry | 0.1424 | 0.1886 | 0.0953 | 0.5514 | 0.1151 | 0.6615 |
| No. SCC | 3 | 2 | 3 | 2 | 1 | 1 |
| Size Largest SCC | 107 | 108 | 156 | 157 | 183 | 183 |
| No. WCC | 1 | 1 | 1 | 1 | 1 | 1 |
| APL (Undirected) | 1.5646 | 1.2586 | 1.4811 | 1.3383 | 1.4217 | 1.2899 |

**Table 1.** IMN vs. ITN: Descriptive Network Statistics. *Note*: SCC: Strongly connected components. WCC: Weakly connected components. APL: Average path length.

| | (1) | (2) | (3) | (4) | (5) |
|---|---|---|---|---|---|
| $\log(\delta_{ij})$ | -1.146*** | -0.795*** | -0.815*** | -0.794*** | -0.820*** |
| CONTIG | 0.511*** | 0.413*** | 0.414*** | 0.411*** | 0.395*** |
| COMLANG | 0.529*** | 0.460*** | 0.465*** | 0.451*** | 0.466*** |
| $\text{PTA}^y$ | 0.434*** | 0.339*** | 0.297*** | 0.286*** | 0.264*** |
| $\log(\text{BIL\_MIG}^y_{ij})$ | | 0.092*** | 0.087*** | 0.088*** | 0.081*** |
| $\log(\text{IN\_CENTR}^y_{ij})$ | | | 0.800*** | | |
| $\log(\text{COMM\_IN}^y_{ij})$ | | | | 0.129*** | 0.324*** |
| $\log(\text{NOTCOMM\_IN}^y_{ij})$ | | | | | 0.060*** |
| No. Obs. | 58812 | 58812 | 58812 | 58812 | 57846 |
| Adjusted $R^2$ | 0.743 | 0.746 | 0.846 | 0.846 | 0.850 |

**Table 2.** Gravity-model estimation with **binary network variables**. Full-sample (pooled) ordinary least-square (OLS) fit. Years $y = 1960, \ldots, 2000$. Dependent variable: logs of total bilateral trade $\tau^y_{ij} = t^y_{ij} + t^y_{ji}$. Country-year dummy variables for importer/exporter effects and constant included. Explanatory variables: See main text. Significance levels: $^{***} = 1\%, ^{**} = 5\%, ^{*} = 10\%$.

| | (1) | (2) | (3) | (4) | (5) |
|---|---|---|---|---|---|
| $\log(\delta_{ij})$ | -1.146*** | -0.795*** | -0.813*** | -0.804*** | -0.799*** |
| CONTIG | 0.511*** | 0.413*** | 0.413*** | 0.429*** | 0.423*** |
| COMLANG | 0.529*** | 0.460*** | 0.469*** | 0.467*** | 0.460*** |
| $\text{PTA}^y$ | 0.434*** | 0.339*** | 0.270*** | 0.293*** | 0.257*** |
| $\log(\text{BIL\_MIG}_{ij}^y)$ | | 0.092*** | 0.086*** | 0.077*** | 0.076*** |
| $\log(\text{IN\_CENTR}_{ij}^y)$ | | | 0.829*** | | |
| $\log(\text{COMM\_IN}_{ij}^y)$ | | | | 0.593*** | 0.443*** |
| $\log(\text{NOTCOMM\_IN}_{ij}^y)$ | | | | | 0.098*** |
| No. Obs. | 58812 | 58812 | 58812 | 57828 | 57828 |
| Adjusted $R^2$ | 0.743 | 0.746 | 0.809 | 0.813 | 0.822 |

**Table 3.** Gravity-model estimation with **weighted network variables**. Full-sample (pooled) ordinary least-square (OLS) fit. Years $y = 1960, \ldots, 2000$. Dependent variable: logs of total bilateral trade $\tau_{ij}^y = t_{ij}^y + t_{ji}^y$. Country-year dummy variables for importer/exporter effects and constant included. Explanatory variables: See main text. Significance levels: $^{***} = 1\%, ^{**} = 5\%, ^{*} = 10\%$.
